# Assessment of sexual dimorphism using 3D CBCT image data among Indians

**DOI:** 10.6026/97320630018231

**Published:** 2022-03-31

**Authors:** Sowmya Hemanthakumar, K Saraswathi Gopal, P Mahesh Kumar

**Affiliations:** 1 Department of Oral Medicine and Radiology, Meenakshi Ammal Dental College & Hospital, Chennai, Tamil Nadu, India

**Keywords:** Cone Beam Computed Tomography, sexual dimorphism, bizygoma, intermaxilla

## Abstract

It is of interest to investigate the use of frontal sinus morphology, bizygomatic and intermaxillary distance for the determination of gender using Cone-Beam Computer Tomography (CBCT). The study population consisted of 75 subjects (35 females and 40 males) with a mean age of 39.25 years (range: 20-70 years), of ethnic group of south-Indian based population. The data was categorized into three age groups of 20-35, 36-50 and ≥ 50 years. All the features and measurements are recorded for each case using CBCT images that were acquired with a CBCT scanner (Planmeca Mid Proface Cone Beam 3D, Helsinki Finland). The data were subjected to a discriminant functional analysis, compared and statistically analyzed. No two persons had the same measurements. Statistically significant differences were found in the frequency of overall metric parameters between the two genders (P < 0.05) except intermaxillary distance (P = -0.034) respectively. These data provide a valuable tool in differentiating gender. It should be noted that bizygomatic distance can significantly improve the gender determination using discriminant analysis. Cone beam computed tomography is a safe procedure with minimal radiation exposure proved to be highly accurate in sinus imaging and provide irreplaceable and precise information about frontal sinus and the whole skull. Measurements showed significant difference except intermaxillary distance and intersinus width among the three age groups. The discriminant analysis showed that the ability of frontal sinus parameters and bizygomatic distance to identify gender with high accuracy.

## Background:

Lois Me Master Bujold stated that "The dead cannot cry out for justice; it is the duty of the living to do so for them"[1]. Identity is the set of physical characteristics, functional or psychic, normal or pathological, that defines an individual. Since time immemorial, human identification has proven to be a basis of civilization and sex identification of unknown individuals. It has always been of paramount importance to the society in forensic sciences [2,3]. The most reliable means of identification include fingerprints, dental comparison, and biological methods such as DNA profiling used in issues such as criminal investigations, insurance settlements, and military proceedings that can be resolved only with the identification [4]. It involves the comparison of ante-mortem radiographs, usually performed for clinical reasons, with post-mortem radiographs taken solely for the identification of specific, individual structures [5]. The frontal sinus is an aeric cavity located within the frontal bone. It develops during the fourth or fifth week of intra uterine life and continues to grow after birth until early adulthood by antero superior pneumatisation of the frontal recess into the bone [6 - check with author]. It contains two chambers which are typically asymmetrical, due to the independent development of each sinus separated by a bone septum [7]. The radiographic pattern uniqueness of frontal sinus among monozygotic twins to every individual has been demonstrated in previous studies. The frontal sinuses radiographs are affluently used in today's forensic medicine for confirmation of personal identity [8] [9]. Therefore, it is important to develop methods using alternate areas of the skeleton to be used for personal identification. It has been described that frontal sinus and zygomatic bones remains undamaged although the skull & other bones may be poorly disfigured in victims [10]. CBCT is well suitable for the investigation of cranio-facial area as it delivers clear images of highly contrasted structures for evaluating bones. A medical imaging technique consisting of X-ray computed tomography where the X-rays are divergent forming a cone is advantages compared with conventional CT [14][15]. The tendency of people is not to keep the conventional radiographs after the end of the illness. However, CBCT, CT scans and MRIs are usually preserved because of their costs [16]. Thus, a combined use of different frontal sinus dimensions, bizygomatic and intermaxillary width helps in precise identification. Therefore, it is of interest to investigate the use of frontal sinus morphology, bizygomatic and intermaxillary distance for the determination of gender using Cone-Beam Computer Tomography (CBCT).

## Materials and Method:

The CBCT images obtained from archives of the oral medicine and radiology department of Meenakshi Ammal Dental College were used in this analysis. The CBCT patient images have been taken for various other purposes included (Orthodontics, Endodontics, Maxillofacial Surgery, ENT and dental implants) were used in this analysis. Forthcoming subjects reported for various other purposes and fulfilled our inclusion criteria were informed about the study and a signed consent in a prescribed form was obtained. The CBCT images were acquired with a CBCT scanner (Planmeca mid Proface Cone Beam 3D, Helsinki Finland). Scanning parameters were 54-90 kv + 5 %, 1-14 mA + 10%, Pulsed, effective 2.4-12 s, 180-240 V/50 Hz of line voltage, 8-15 mA of line current. The CBCT volume data were reconstructed using the CBCT software (PlanmecaRomexis). The CBCT images of 150 frontal sinuses of 75 individuals (35 females and 40 males), aged above 20 years (ranges: 20-70) were examined. The intention of limiting the sample to young adults was based on fact that frontal sinuses complete their development by approximately 20 years and remains stable. The walls become thin and appear to be larger in the old people. Patients were divided into three age groups of 20-35 years, 36-50 years and ≥50 years. Inclusion criteria are normal healthy individuals of age 20 years and above. Exclusion criteria are patients with disease or pathologic conditions involving the frontal and maxillary sinus including developmental abnormalities affecting normal anatomy of frontal and maxillary sinus and images with artifacts. The study was approved by the Ethics Research Committee at the University. CBCT images were evaluated to examine and classify the variations in the pattern of frontal sinus dimensions, bizygomatic and intermaxillary width as observed on the images. The measurement was taken after going through in coronal and axial view (Table 1 - see PDF).

## Statistical analysis:

The Normality tests Kolmogorov-Smirnov and Shapiro-Wilks tests results reveal that the variables follow a normal distribution. Therefore, parametric methods are applied to analyse the data. The mean values between genders independent samples t-test is applied for comparison. Chi-Square test is applied to compare proportions between genders. Fisher’s exact test is used if the expected cell frequency is less than five. Discriminant analysis is performed to classify the gender. SPSS (IBM SPSS Statistics for Windows, Version 22.0, Armonk, NY: IBM Corp. Released 2013) is used to analyse the data. Significance level is fixed as 5% (α = 0.05).

## Results & Discussion:

Statistically significant differences (Table 2 - see PDF) were found in the frequency of total sinus width, total width of individual sinuses, intersinus width, distance highest points of two sinuses, distance highest points of right sinus and lateral limit, distance highest points of left sinus and lateral limit, bizygomatic distance between the two sexes (P<0.05) except intermaxillary distance (P Value - 0.034). All frontal sinus and bizygomatic distance measurements showed a statistically significant gender difference (except for the intermaxillary distance). Discriminant functional analysis was applied to classify the overall gender. The four variables with standardized function coefficients of 0.49 in total sinus width, 0.35 in left maximum height, 0.622 in left anteroposterior diameter and 0.479 in distance highest points of right sinus and lateral limit were identified as best discriminating variables by stepwise procedure. Both canonical and fisher’s linear discriminant equation were developed in this analysis. The left anteroposterior diameter was the best discriminating variable (associated with the largest standardized coefficient) followed by total sinus width, distance between the highest point of right sinus and lateral limit and left maximum height obtained in this method is given in Table 3 to 5(see PDF).

Discriminant functional analysis was applied to classify the gender (Table 6 - see PDF). The three variables with standardized function coefficients of 0.882 in total sinus width, 0.47 in left maximum height, 0.45 in left anteroposterior diameter were identified as best discriminating variables by stepwise procedure. Both canonical and fisher's linear discriminant equation were used. The age group of 20-35 years, total sinus width (0.882) was the best discriminating variable (associated with the largest standardized coefficient) followed by left maximum height (0.470) and left anteroposterior diameter (0.456) as seen in Table 7(see PDF).

Discriminant functional analysis was applied to classify the gender. The two variables with standardized function coefficients of 0.623 in left anteroposterior diameter, 0.693 in left maximum height, were identified as best discriminating variables by stepwise procedure. Both canonical and fisher's linear discriminant equation were developed. The age group of 36-50 years, the left maximum width (0.693) was the best discriminating variable (associated with the largest standardized coefficient) followed by left anteroposterior diameter (0.623) as seen in Table 8(see PDF). To classify the gender discriminant functional analysis was applied. The five variables with standardized function coefficients of 0.793 in left maxillary height,0.965 in left anteroposterior diameter, 1.121 in right maxillary height, 1.226 in distance highest points of two sinuses and 1.552 in distance highest points of right sinus and lateral limit were identified as best discriminating variables by stepwise procedure. Both canonical and fisher's linear discriminant equation were developed. In this model the age group ≥ 50 years, the distance between highest points of right sinus and lateral limit (1.552) was the best discriminating variable (associated with the largest standardized coefficient) followed by distance highest points of two sinuses (1.226), right maximum height (1.121), left anteroposterior diameter (0.965) and left maximum height(0.793) as seen in (Table 9 - see PDF). Identification using skull measurements remains the most widely used method for personal identification. In the present study, CBCT was utilized for skull imaging. CBCT produces three-dimensional information on the facial skeleton and teeth are increasingly being used in many of the dental specialties. So CBCT produced several advantages for forensic imaging. It has practical advantages of relatively small size, portability and low cost and technical advantages of good spatial resolution and metal artifact reduction [17].

There are considerable variations in the shape, capacity, and symmetry of the frontal sinuses. Data states that 3 individuals (3%) had bilateral absence of the frontal sinuses was in agreement with the studies [18] in 2016 (Iran) including 2% in males and 3.5% in females in 2016 [19] where bilateral absence of the frontal sinus was observed in 7 individuals (14% of the study group). Out of them 2 are females (9.52%) and 5 are male (17.24%). These findings are considered different from those [20] who studied antero-posterior plane radiographs of skull of 300 Indian population and found absence of frontal sinus in 4.63% of cases; 1.3% of males and 3.33% of females.

In 2011 data in [21] reported lower incidence than the present study. In 1977 (Germany) [22] and [23] (Turkey) in 2003; while it was less than the finding [24] in 1987 (Japan), [25] in 1972 (Alaskan Eskimo) [26] (Canadian Eskimo). Most of these studies indicate a greater frequency among females than males. This is similar to the findings in this study. Our findings are different from the results [27] in 2010, [28] in 2002, [29] in 2011 and [30] in 2010 studies. Race, populations, technique, methodology, climate and geographical conditions, inflammation and mechanical stress can be mentioned as a few factors, which might have contributed to the observed difference. All measurements had higher values in the males and the differences were significant (p <0.05). Measurements were evaluated for each sex on right and left sides and they were different in both sexes. Furthermore, larger right side was demonstrated with the mean values of 40 males were 27.27 and 27.50 and in 35 females were 15.90 and 15.71 respectively, which was in agreement to the study [19] in 2016 where larger right side was demonstrated in 20 subjects (40% of the study group), which is in contrast to the study conducted [31] in 2007, [32] in 2008 and [33] in 2010 who found right frontal sinus smaller than the left one in their studied populations.

The presence of one side larger than the other is due to their independent development. Although not always statistically significant, the frontal sinuses were generally larger in males than females in previous studies [34] in 1970,[24] in 1987,[35] in 1997, [36] in 2000, except in the study [26] on the Canadian Eskimo population who reported that the frontal sinuses were dimensions were larger in females. The absence of scalloping feature of frontal sinus is distinctive among the studied sample (82.7% for right side and 84% for the left side). Other studies revealed (22.5 for right side and 25% for the left side) [30] in 2010, and [29] in 2011 in only 4% of studied sample. The number of scalloping in men was higher than women in this study and the difference did not reach a statistically significant level (p < 0.05). This study was in agreement [18] (Iran) in 2016 but in contrast with the viewpoint [37] in 2004 in which the number of frontal sinuses scalloping in women is claimed to be more than men. This could potentially be the effect of race, methodology or an inadequate sample size. Sinus Septum was found in all the subjects included in the study (100%of the study group). This finding is also found in 2016 [19], [30] in 2010 who studied frontal sinus in Iraq population using spiral CT scanning. This finding does not agree with [27] in 2010 and [20] in 2014 who remarked that there is no sinus septumin 3.8% and 1.3% of their studied group of population, respectively. The mean maximum width of right and left frontal sinus in our study of males were 27.27±4.19 and 27.50±3.68 and in 35 females they were 15.90±3.25 and 15.71±3.22 respectively which is similar to the reported means [18] in 2016, [19] in 2016, [29] in 2011 and [30] in 2010, [28] in 2002, but different [27] in 2010. The mean maximum height of right and left frontal sinus in our population of males were 27.98±3.63 and 29.33±3.67; females were 15.95±3.62 and 16.11±3.12 respectively is less than the mean height reported [18] in 2016, [19] in 2016, [29] in 2011, [30] in 2010, yet our findings are similar [38] in 2010, and [39] in 2013, where mean height is significantly greater in males than females. Mean antero-posterior diameter of right and left frontal sinus in the population of mean values of males were 16.99±2.49 and 16.94±2.22 and females were 9.01±2.05 and 8.53±1.49 respectively which is similar to the results [18] in 2016, [19] and [29] in 2011, [30] in 2010, but different from the results [38] in 2010 and [39] in 2013. The mean inter sinus width in males were 1.46 and females were 1.24. Other studies have not stated this difference. Therefore, we cannot do any comparison with those populations. We did not find a significant difference in the number of complete septa between the two sexes (P-Value 0.051). This might be due to inadequate sample size or this parameter is simply not useful for sex determination. We did not find any studies addressing this comparison between the two sexes.

The mean total sinus width in males were 55.84±7.88 and females were 32.86±5.58 respectively and this is similar to the results [18] in 2016 between the age group of 20-34 years with p-value of 0.001 but not in 35-49 and more than 50 years of age, [19][30] in 2010. The mean distance between highest points of two frontal sinuses of males were 13.70±4.30 and in females were 6.63±2.78 respectively and this is similar to the study [19] in 2016 where males have 13.23±4.04 and females have 15.18±9.47 [30] in 2010. The mean distance highest points of right sinus and lateral limit between two frontal sinuses of males were 23.18±5.85 and in females were 11.63±6.64 respectively. This study is in accordance [30] in 2010. The mean distance highest points of left sinus and lateral limit between two frontal sinuses of males were 24.11±5.81 and in females were 11.00±6.14 respectively and it is similar to the study [30] in 2010. In our study, the overall average dimensions of each parameter were statistically greater for males compare with females. The mean ± SD of bizygomatic distance in male was 97.38± 3.59mm & in female was 93.94±4.32 mm & the total average (M+F) was 144.35±5.75 mm which is significant statistically (p<0.01) and can be a strong parameter used for gender determination. Frontal sinus measurements were used to discriminate between males and females using functional analysis. The left anteroposterior diameter was the best discriminating variable (associated with the largest standard coefficient), followed by total sinus width, then the distance between highest point of right sinus and lateral limit, left maximum width, right and left maximum height, distance highest points of two sinuses in this model. Hence, frontal sinus measurements can be taken using discriminant analysis, whereas a study [30] in 2010 has done a functional analysis to discriminate between males and females frontal sinus measurements resulted in a model with an overall accuracy of 76.95 and Wilk's lambda of 0.75.

## Conclusion:

Frontal sinus measures and non-metric characteristics are unique for individuals to help for personal identification in forensic practice. Unique sinus morphology and anatomy also have significance for cranio-plasty and sinus surgery. Data shows that males have larger frontal sinus than females. Thus, it can be used also for gender differentiation and a high precision of gender determination was found to be for the left antero-posterior diameter (Depth). Cone beam computerized tomography (CBCT) with technique involving low dose proved to be uncomplicated, expeditious, and precise and it is a producible method for frontal sinus examination. It proved to reduce the error rates to give more accurate measurements and descriptors than other methods used for frontal sinus examination. Therefore, it is concluded that the measured dimensions of male were found to be larger than those of female. This difference was statistically significant for Bizygomatic distance (p<0.005) except intermaxillary distance (P value 0.336). The results obtained were comparable to the previous studies and it can be used as an aid in forensic anthropology for gender determination to some extent. Hence, we conclude that Cone beam Computed Tomography measurements of frontal sinus and bizygomatic distance are useful to support gender determination in forensic medicine when other methods are inconclusive.

## Limitations:

There are neither standardized measurements of the frontal sinus nor known error rates of every technique. Ante-mortem frontal sinus imaging is not routine in many countries. These short comings make frontal sinus method for identification still inadmissible in the court.

## Future Perspectives:

This study focused mainly on the evaluation of linear measurements of frontal sinus, bizygomatic and intermaxillary distance. However, volumetric assessment of frontal sinus was not assessed. Studies related to the establishment of a volumetric approach of frontal sinus human identification through usage of 3D models obtained from CBCT exams will be assessed in future investigations.
